# Iodixanol Density Gradient Preparation in University of Wisconsin Solution for Porcine Islet Purification

**DOI:** 10.1100/tsw.2003.107

**Published:** 2003-12-01

**Authors:** Michael P.M. Van der Burg, John M. Graham

**Affiliations:** Department of Surgery K6-50, Leiden University Medical Centre, Leiden, The Netherlands

**Keywords:** Islets of Langerhans, purification, iodixanol, OptiPrep™, University of Wisconsin solution, density barrier, transplantation, diabetes, implants, pig, isolation

## Abstract

Previously published as Graham, J.M. (2002) Purification of Islets of Langerhans from porcine pancreas. TheScientificWorldJOURNAL 2, 1657–1661. ISSN 1537-744X; DOI 10.1100/tsw.2002.847.Generally, prior to the purification of isolated pancreatic islets, the collagenase-digested tissue is incubated in the University of Wisconsin solution (UWS; ~320 mOsm) for osmotic stabilization to preserve or improve the density differences between islets and acinar fragments. The adverse effects arising from the subsequent pelleting and resuspension of the islets in a second, different (often highly hyperosmotic) purification solution are avoided in the protocol described here; preparation of the purification medium is simply achieved by mixing the UWS preincubated islets with a second UWS containing the inert impermeant iodixanol. Flotation of the islets isolated from juvenile porcine pancreases through this mildly hypertonic (~380 mOsm) gradient of iodixanol-UWS achieves a much higher recovery of islets of an improved viability than the customary method using a Ficoll gradient. The method has been extended to human islet purification.

**METHOD TYPE:** extraction, isolation, purification and separation

## INTRODUCTION

Although this protocol has been developed for the isolation of islets from porcine pancreas[1,2,3], preliminary reports show that it can be successfully adapted to tissue from humans[4,5]. Optimal recoveries may vary with the species, with donor factors like age, and tissue handling procedures, and may therefore require minor adjustments to the density of the gradient solutions.

The aim of this protocol is to describe a procedure for islet purification rather than to provide a detailed account of the method of islet isolation. It is based on an islet isolation method for the juvenile pig pancreas using the University of Wisconsin solution (UWS) as the medium for both collagenase digestion of the tissue at 37°C and for all postdigestion operations (mechanical dispersion, filtration, etc.) carried out at 2–4°C[1,2].

Some workers may prefer to restrict the use of UWS to the "cold" steps (it may perhaps be slightly cytotoxic at 37°C, or it may inhibit digestion in other species); in which case the digestion should be carried out in a physiological salt-based solution such as Hanks Balanced Salt Solution (HBSS) or perhaps a tissue culture medium (see Note 1). If such a medium is also used for the preparation of the density gradient solutions, modifications will need to be made to the volumes of OptiPrep™ and medium because these culture media have a lower density than that of UWS and also may affect tissue density differently (see Notes 2 and 3).

The protocol uses a Working Solution containing 30% w/v iodixanol (osmolarity approx. 500 mOsm) produced by mixing OptiPrep™ (Axis-Shield, Oslo, Norway; a 60% w/v solution of iodixanol in water, $\rho$ = 1.32 g/ml) with an equal volume of double-strength UWS ($UWS_{2x}$). Gradient solutions are subsequently prepared by diluting the Working Solution with standard (1x) UWS (see Note 2). The protocol uses 50-ml conical centrifuge tubes in which the islet suspension (in UWS) is adjusted to $\rho$ = 1.10 g/ml (osmolarity approx. 380 mOsm) by mixing with the Working Solution and layered beneath a low-density barrier ($\rho$ = 1.09 g/ml) and a top layer of UWS. During centrifugation, the islets float to the interface between the UWS and the $\rho$ = 1.09 g/ml barrier, while the acinar tissue remains in the load zone.

## MATERIALS AND EQUIPMENT

- OptiPrep™ (shake gently before use; obtained from Lucron Bioproducts BV, Gennep, Netherlands).
- Diluent for OptiPrep™: double-strength UWS ($UWS_{2x}$). The reagents for the preparation of $UWS_{2x}$ were obtained from Sigma-Aldrich (Zwijndrecht, Netherlands) unless stated otherwise. To prepare 2 l, dissolve 143.3 g of lactobionic acid (200 mM; lactobionic acid 97%, Sigma 15351-6) in 1250 ml of sterile pyrogen-free water, then dissolve 4.00 g NaOH (50 mM; sodium hydroxide ≥98%, Sigma S-8045), and adjust to pH 7.0 with 5 M KOH (potassium hydroxide ≥85%, Sigma P-5958) before adding the following in the order given (see Note 4):
  - 13.6 g $KH_2PO_4$ (50 mM; potassium dihydrogen phosphate anhydrous 99.7%, Sigma P-0662)
  - 2.40 g $MgSO_4$ (10 mM; magnesium sulfate anhydrous, Sigma M-2643)
  - 71.3 g raffinose (60 mM; D-raffinose pentahydrate 98%, Sigma 20667-9)
  - 0.27 g allopurinol (1 mM; allopurinol, Sigma A-8003; see Note 5)
  - 3.68 g glutathione [reduced] (6 mM; L-glutathione reduced ≥98%, Sigma G-6013)
  - 5.34 g adenosine (10 mM; adenosine, Sigma A-4036)
  - 200 g pentastarch (100 g/l; pentastarch powder, hydroxyethylstarch approx. 250 K Dalton, code no: E9701707, Fresenius Pharma, Linz, Austria; see Note 6)

Adjust with 5 M KOH to pH 7.4 and make up to 2 l. Density is 1.092 g/ml (1.0915–1.0925 g/ml). Filter sterilize and store the solution at 2–4°C.

- Diluent for gradient solutions: UWS (used also as the general isolation solution). Prepare the UWS by diluting $UWS_{2x}$ with an equal volume of sterile distilled water. Check the pH (7.4) of the solution, filter sterilize, and store at 2–4°C. Density is 1.046 g/ml, and osmolarity is 320 ± 5 mOsm (measured by depression of freezing point). See Notes 6 and 7.
- Polypropylene 50-ml screw-cap conical centrifuge tubes (Falcon®, Becton Dickinson Labware); sterile polystyrene media bottles and bottle-top CA (cellulose acetate) –0.22 µm filter systems (Corning, Schiphol Rijk, Netherlands).
- Syringes (20 ml, luer lock) and blunt wide-bore metal cannulas (approx. 2.0–2.2 × 120 mm) for overlayering and islet harvesting.
- Low-speed (temperature-controlled) centrifuge with swing-out rotor (Beckman J-6M/E centrifuge with JS3.0 rotor; Beckman Instruments, Palo Alto, CA).
- Pyrogen-free glass 5-l Erlenmeyer flasks, 1-/2-l beakers, 0.5-/1-/2-l graduated cylinders, stirring bar and magnetic stir plate.
- Optional: Paar DMA 35N density meter (Anton Paar Gmbh, Graz, Austria; obtained from This Sci., Sliedrecht, Netherlands) for quality control measurements of the density of the solutions.

## METHODS

(Summarized in Fig. 1)

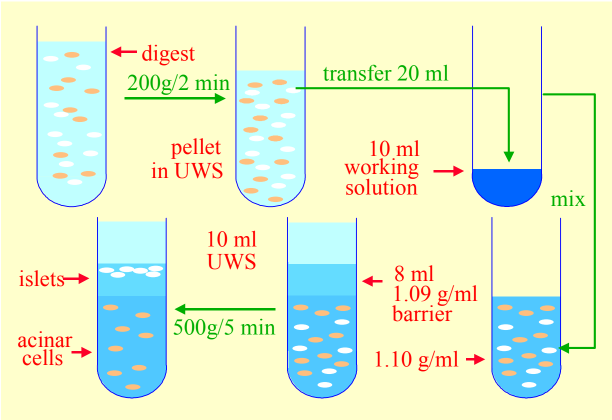

**FIGURE 1.** Islet purification flow diagram.

1. Prepare the Working Solution (ρ = 1.206 g/ml; osmolarity ~500 mOsm) by mixing equal volumes of OptiPrep™ and $UWS_{2x}$ and transfer 10-ml aliquots to 50-ml conical centrifuge tubes. Keep these at 2–4°C.
2. Prepare the low-density barrier solution (ρ = 1.090 g/ml) by mixing 10 ml of the Working Solution with 26.4 ml of UWS and keep at 2–4 °C (see Notes 8 and 9).
3. Digest the pancreatic tissue with collagenase in UWS (or other chosen medium) at 36–37°C, then carry out all subsequent operations (mechanical dispersion, filtering etc) in UWS at 2–4°C.

4. Centrifuge the digest for 2 min at 200*g* at 4°C and gently resuspend the pellet in UWS and make up to volume (a multiple of 20 ml) with this medium (e.g., 10 ml of packed tissue pellet in a total volume of 80 ml).
5. Transfer 20 ml of digest suspension into each of the prepared centrifuge tubes containing 10 ml of Working Solution and mix rapidly but gently by repeated inversion or pouring repeatedly between two centrifuge tubes.
6. Layer 8 ml of the low-density barrier solution over the suspension and top up the tube with 10 ml of (1x) UWS.
7. Centrifuge at 500*g* for 5 min at 4°C. The islets band at the top interface; acinar tissue remains in the load zone (see Fig. 1 and Notes 10–12).
8. Harvest the islets using a syringe and wide-bore metal cannula; dilute with 3–4x the volume of (1x) UWS and pellet at 200*g* for 4 min.

## Notes

1. If a medium such as HBSS or RPMI is used for the postdigestion steps, the tissue should be preincubated in cold UWS for 60 min before addition of the Working Solution. The gradient however may require significant adjustment of density and perhaps osmolality[2].
2. $UWS_{2x}$ has a density of 1.092 g/ml. Double-strength HBSS or RPMI have a lower density (approx 1.012 g/ml), consequently the amount of single-strength medium required to produce solutions of the appropriate density will require modifying (see Notes 3 and 8).
3. See Ref. 6 for more information about preparing density gradient solutions for mammalian cells.
4. Neutralization of the lactobionic acid should be carried out slowly and carefully.
5. Allopurinol is kept at the same concentration as in UWS (1x) as higher concentrations are difficult to dissolve.
6. Contact the first author (e-mail: burg@lumc.nl) or Mr. B. Henriksen (Axis-Shield PoC; fax: +47 22 04 20 01; e-mail: bjh@no.axis-shield.com) regarding commercial sources of pentastarch powder and UWS (1x; ViaSpan) and ordering information.
7. Note that by diluting $UWS_{2x}$ with an equal volume of water, the allopurinol concentration will be half that normally in UWS (1x). Alternatively UWS may be purchased commercially or it can be prepared using half the concentration of the reagents in $UWS_{2x}$ (except allopurinol which should be at the same concentration).
8. It may be necessary to modulate the density of this layer[2] according to the isolation method that is used or if islets are purified from other species. Table 1 gives the volumes of UWS and Working Solution required to produce solutions of different densities.
9. It may be an advantage to produce the barrier solution in RPMI, this can act as a preliminary means of washing the islets free from UWS, as they float to the upper interface. Good results have been obtained with barrier solutions prepared by diluting OptiPrep™ with RPMI or RPMI containing 10% serum: 3.2 ml of OptiPrep™ and 8.8 ml of RPMI gives a solution of $\rho = 1.090$ g/ml; if RPMI containing 10% serum is used the density is approx 1.092 g/ml.
10. Unacceptable levels of acinar tissue contamination in the islet layer normally imply that the density of the barrier layer is too high and should be reduced.
11. As shown in Table 2 the yield, viability, purity, and resistance to fragmentation (size) of the islets purified by this technique show a marked improvement in all four parameters compared to those purified by the routine ficoll technique (Ficoll-Na-diatrizoate; Histopaque®, Sigma, St. Louis, Mo); indeed the yield of islets from the digest is virtually doubled[1]. These results were corroborated in the Minneapolis center[3].

**TABLE 1**
**Density of Solutions Prepared from Mixing 10 ml of Working Solution (1.206 g/ml) and Different Volumes of UWS**

| UWS (ml) | Density (g/ml) |
|---|---|
| 22.65 | 1.095 |
| 26.36 | 1.090 |
| 31.03 | 1.085 |
| 37.06 | 1.080 |
| 45.17 | 1.075 |

**TABLE 2**
**Comparison of Islet Purification Methods Following Collagenase Digestion of Juvenile Porcine Pancreases**

| Purification Method | Yield (% of digest) | Viability (%) | Purity (% of cells) | Size (μm) |
|---|---|---|---|---|
| OptiPrep™ | 105 ± 16 | 75 ± 3 | 86 ± 3 | 96 ± 11 |
| Ficoll | 58 ± 10 | 54 ± 4 | 61 ± 9 | 82 ± 5 |

Data are means ± SE.

12. Already during the preparation (layering) of the gradients in the conical centrifuge tubes, islets can be observed migrating upwards. Thus, possible trauma from centrifugation may perhaps be minimized by reducing the centrifugal force. Recent work using the Cobe centrifuge for purification suggests that changing the RCF from 800 to 100*g* improves the outcome[7].

## ACKNOWLEDGMENTS

The authors and TheScientificWorldJOURNAL wish to thank Axis-Shield PoC, AS, Oslo, Norway for their kind permission to adapt OptiPrep™ Application Sheet C15 in the preparation of this Protocol Article.

## References

1. Van der Burg, M.P.M., Basir, I., and Bouwman, E. (1998) No porcine islet loss during density gradient purification in a novel iodixanol in University of Wisconsin solution. *Transplant. Proc*. **30**, 362–363.
2. Van der Burg, M.P.M., Basir, I., Zwaan, R.P., and Bouwman, E. (1998) Porcine islet preservation during isolation in University of Wisconsin solution. *Transplant. Proc*. **30**, 360–361.
3. Matsumoto, S., Zhang, H.J., Gilmore, T., van der Burg, M.P.M., Sutherland, D.E.R., and Hering, B.J. (1998) Large scale isopycnic islet purification utilizing non-toxic, endotoxin-free media facilitate immediate single-donor pig islet allograft function. *Transplantation* **66**, S30.
4. Van der Burg, M.P.M., Ranuncoli, A., Molano, R., Kirlew, T., Ringers, J., Bouwman, E., and Ricordi, C. (1998) Efficacy of the novel iodixanol-UWS density gradient for human islet purification. *Acta Diabetol.* **35**, 247.

5. Van der Burg, M.P.M., Ranuncoli, A., Molano, R., Kirlew, T., Ringers, J., Bouwman, E., Terpstra, O.T., and Ricordi, C. (1999) OptiPrep for human islet purification. *Cell Transplant.* **8**, 184.
6. Graham, J.M. (2002) OptiPrep™ density gradient solutions for mammalian organelles. *TheScientificWorldJOURNAL* **2,** 1440–1443.
7. Shibata, S., Sageshima, J., Hiraoka, K., Zhang, H., Koyama, K., Sutherland, D.E.R., and Hering, B.J. (2001) Low-speed isopycnic islet separation is effective and yields islets with superior quantity and quality. *Acta Chir. Austriaca* **33(Suppl. 174),** 62.
